# Targeted analysis of genomic regions enriched in African ancestry reveals novel classical HLA alleles associated with asthma in Southwestern Europeans

**DOI:** 10.1038/s41598-021-02893-w

**Published:** 2021-12-08

**Authors:** Eva Suarez-Pajes, Claudio Díaz-García, Héctor Rodríguez-Pérez, Jose M. Lorenzo-Salazar, Itahisa Marcelino-Rodríguez, Almudena Corrales, Xiuwen Zheng, Ariel Callero, Eva Perez-Rodriguez, Jose C. Garcia-Robaina, Rafaela González-Montelongo, Carlos Flores, Beatriz Guillen-Guio

**Affiliations:** 1 Research Unit, Hospital Universitario Nuestra Señora de Candelaria, Universidad de La Laguna, Santa Cruz de Tenerife, Spain; 2 Genomics Division, Instituto Tecnológico Y de Energías Renovables (ITER), Santa Cruz de Tenerife, Spain; 3 CIBER de Enfermedades Respiratorias, Instituto de Salud Carlos III, Madrid, Spain; 4 Department of Biostatistics, University of Washington, Seattle, WA USA; 5 Allergy Unit, Hospital Universitario N.S. de Candelaria, Santa Cruz de Tenerife, Spain; 6 Department of Health Sciences, University of Leicester, Leicester, UK

**Keywords:** Genetics, Genetic association study, Asthma

## Abstract

Despite asthma has a considerable genetic component, an important proportion of genetic risks remain unknown, especially for non-European populations. Canary Islanders have the largest African genetic ancestry observed among Southwestern Europeans and the highest asthma prevalence in Spain. Here we examined broad chromosomal regions previously associated with an excess of African genetic ancestry in Canary Islanders, with the aim of identifying novel risk variants associated with asthma susceptibility. In a two-stage cases-control study, we revealed a variant within *HLA-DQB1* significantly associated with asthma risk (rs1049213, meta-analysis *p* = 1.30 × 10^–7^, OR [95% CI] = 1.74 [1.41–2.13]) previously associated with asthma and broad allergic phenotype. Subsequent fine-mapping analyses of classical HLA alleles revealed a novel allele significantly associated with asthma protection (HLA-DQA1*01:02, meta-analysis *p* = 3.98 × 10^–4^, OR [95% CI] = 0.64 [0.50–0.82]) that had been linked to infectious and autoimmune diseases, and peanut allergy. HLA haplotype analyses revealed a novel haplotype DQA1*01:02-DQB1*06:04 conferring asthma protection (meta-analysis *p* = 4.71 × 10^–4^, OR [95% CI] = 0.47 [0.29– 0.73]).

## Introduction

Asthma is a complex respiratory disease characterized by reversible airflow obstruction and chronic inflammation of the lower respiratory tract, usually linked to allergic and atopic manifestations^1^. Asthma has an increasing prevalence, affecting more than 300 million people worldwide^2,3^, which represents a high health care cost. In Europe, the economic burden of asthma in adults is estimated to account for over €19 billion per year^4^. This global health problem is partly because of the heterogeneity of the disease that makes both its clinical management and research challenging. In this sense, asthma and the allergic traits are strongly conditioned by genetic factors, with heritability estimates ranging between 35 and 95%^5^. Several genome-wide association studies (GWAS) have revealed several asthma risk genes, including those encoding the ORMDL Sphingolipid Biosynthesis Regulator 3 (*ORMDL3*), Gasdermin B (*GSDMB*), and Cadherin Related Family Member 3 (*CDHR3*), as well as a number of genes related to innate immunity and immunoregulation^6^.

Despite the efforts made by large consortia, less than 5% of asthma variability can be explained by the genetic risk variants revealed to date^7^. Among other reasons, this could be because most of the previous genetic association studies have focused on patients of European ancestry^8–10^. Therefore, other genetic risks that are more frequent in other ethnicities could remain unknown. The key implications of ancestry in asthma risks have been evidenced in previous studies in non-European admixed populations that revealed novel gene risks for asthma in African American and Latino populations^10–14^. However, although in recent years the number of genetic studies in admixed populations has increased considerably, the statistical power in many of them has not been sufficient due to the large sample sizes required by GWAS to overcome the stringent significance penalties, making screening for asthma-related genes in admixed populations still a challenge^10^.

In this sense, studies leveraging local genetic ancestry analyses constitute an alternative to further disentangle the genetics underlying asthma in recently-admixed populations^11,15–19^, given that they attain better study power using more limited sample sizes^20^. Briefly, the genome of admixed individuals is constituted by chromosomal segments from their parental populations. The average proportion with which each parental group contributes to the genome of an admixed individual is known as global genetic ancestry, whereas the local genetic ancestry is defined as the ancestry proportion of each particular locus. Studies such as admixture mapping studies allow to reveal genomic regions where local ancestry correlates with disease risk, and the main challenge remains in the subsequent fine mapping studies of these regions to identify the causal variants^17,21^.

We previously assessed the local ancestry estimates in an admixed southwestern European population, the Canary Islands population (Spain), revealing the largest African genetic ancestry in Europe, with average proportions of 22.0% of North African (NAF) ancestry and 3.0% of Sub-Saharan African (SSA)^22^. Additionally, we identified five genomic regions showing an excess of African ancestry in this population within chromosomes 2, 3 (two regions), 6, and 13. These regions were enriched in genes linked to respiratory diseases, including asthma^22^, which is not surprising since the Canary Islanders have the highest prevalence of asthma in Spain^23,24^. As a matter of fact, we recently performed an admixture mapping of asthma in this population, revealing a novel locus associated with asthma risk^16^.

Based on the previous evidence, we hypothesized that the targeted screening of the five genomic regions from the current Canary Islander population that were found to be enriched in African alleles could reveal novel asthma risks. To test this possibility, we conducted a two-stage association study focused on the regions of interest in Canary Islanders, followed by a fine-mapping study of the highly variable human leukocyte antigen (HLA) region.

## Results

After quality controls, 930 Canary Islanders from stage 1 (313 cases and 617 controls) and 557 from stage 2 (251 cases and 306 controls) remained in the study. Detailed information of these participants can be found in Table 1. Association testing focused on a total of 140,955 imputed genetic variants located on the five loci enriched in African ancestry in the Canary Islands population (2q21.2-q22.3, 3p25.3, 3q26.32, 6p22.3-p21.32, and 13q21.1-q21.33). The number of variants of each region can be found in Supplementary Table S1. After meta-analysis of results from the two stages, the variant rs1049213 from 6p22.3-p21.32 was significantly associated with asthma risk (OR [95%CI] = 1.74 [1.42–2.14]; *p* = 1.30 × 10^–7^) (Table 2). This variant is in the 3′untranslated region (3′ UTR) of the Major Histocompatibility Complex (MHC), Class II, DQ Beta 1 gene (*HLA-DQB1*) (Supplementary Figure S1). We accessed public GWAS data available at Open Target Genetics^25^ and found that this single nucleotide polymorphism (SNP) has been associated with asthma (*p*-value = 1.13 × 10^–38^, OR = 1.12) (http://www.nealelab.is/uk-biobank/) and broad allergic phenotype^26^ before. No significant variants were identified in the other regions of interest on chromosomes 2, 3, and 13.

**Table 1:** Demographic and clinical characteristics of the individuals included in the study.

	Stage 1			Stage 2			Comparison of case data sets
	Cases	Controls	*p-value*	Cases	Controls	*p-value*	*p-value*
**N**	313	617	NA	251	306	NA	NA
**Sex**							
Male	111/313 (35.5%)	311/617 (50.4%)	2.1 × 10^–5^	73/251 (29.1%)	126/306 (41.2%)	4.0 × 10^–3^	3.2 × 10^–4^
Female	202/313 (64.5%)	306/617 (49.6%)	2.1 × 10^–5^	178/251 (70.9%)	180/306 (58.8%)	4.0 × 10^–3^	
Age, yr	31.2 ± 13.5	52.5 ± 15.6	2.2 × 10^–16^	32.1 ± 17.3	42.0 ± 11.2	1.0 × 10^–16^	2.2 × 10^–16^
BMI	25.6 ± 7.1	27.2 ± 4.8	5.2 × 10^–7^	25.0 ± 5.7	26.3 ± 4.3	0.02	0.01
**Smokers**							
Non-smoker	161/210 (76.7%)	NA	NA	170/218 (78.0%)	NA	NA	0.83
Ever-smoker	49/210 (23.3%)	NA	NA	48/218 (22.0%)	NA	NA	
**Clinical features**							
Age at diagnosis, yr	12.2 ± 9.5	NA	NA	20.5 ± 17.0	NA	NA	5.6 × 10^–7^
Specific IgE, %positive	177/217 (81.6%)	NA	NA	111/251 (44.2%)	NA	NA	2.70 × 10^–16^
Skin prick test, %	232/311 (74.6%)	NA	NA	179/251 (71.3%)	NA	NA	0.43

Age and body mass index (BMI) values are represented by means ± SD; N = no. of participants; NA = not available. Statistical differences were obtained by Chi-squared tests for sex, smokers, specific IgE, and skin prick test; and by Wilcoxon tests for age, BMI, and age at diagnosis.

**Table 2:** Ten most significant variants after the meta-analysis of the targeted association. Association results from the 6p22.3-p21.32 region for stage 1, stage 2, and meta-analysis.

rs	Position	Genes	Ref/EA	Stage 1			Stage 2			Meta-analysis	
				*p value*	MAF	OR (95%)	*p value*	MAF	OR (95%)	*p value*	OR (95%)
**rs1049213**	**32,627,773**	***HLA-DQB1***	**A/G**	**9.79 × 10**^**–5**^	**0.24**	**1.74 (1.32–2.30)**	**3.67 × 10**^**–4**^	**0.22**	**1.74 (1.28–2.35)**	**1.30 × 10**^**–7**^	**1.74 (1.42–2.14)**
rs3129782	32,648,719	*HLA-DQB1*,* HLA-DQA2*	G/A	4.80 × 10^–4^	0.25	1.64 (1.24–2.16)	1.21 × 10^–3^	0.22	1.63 (1.21–2.19)	1.92 × 10^–6^	1.63 (1.33–2.00)
rs9273410	32,627,250	*HLA-DQB1*	C/A	4.34 × 10^–5^	0.47	1.62 (1.28–2.04)	0.016	0.47	1.35 (1.06–1.73)	3.73 × 10^–6^	1.49 (1.26–1.76)
rs2442719	31,320,538	*HLA-C*,* HLA-B*	C/T	1.31 × 10^–4^	0.39	1.57 (1.25–1.98)	0.018	0.40	1.35 (1.05–1.72)	1.06 × 10^–5^	1.46 (1.24–1.73)
rs9273400	32,627,128	*HLA-DQB1*	C/T	1.55 × 10^–4^	0.48	1.55 (1.24–1.95)	0.016	0.48	1.34 (1.06–1.70)	1.10 × 10^–5^	1.45 (1.23–1.71)
rs202092176	32,596,429	*HLA-DRB1*,* HLA-DQA1*	C/T	2.00 × 10^–4^	0.15	0.53 (0.37–0.74)	0.017	0.12	0.63 (0.43–0.92)	1.27 × 10^–5^	0.57 (0.44–0.73)
rs9273404	32,627,160	*HLA-DQB1*	A/G	1.30 × 10^–4^	0.49	1.55 (1.24–1.95)	0.027	0.50	1.31 (1.03–1.67)	1.66 × 10^–5^	1.43 (1.22–1.69)
rs9272679	32,608,931	*HLA-DQA1*	T/C	1.24 × 10^–4^	0.43	1.59 (1.25–2.01)	0.037	0.44	1.30 (1.02–1.67)	2.40 × 10^–5^	1.45 (1.22–1.72)
rs9273338	32,623,393	*HLA-DQA1, HLA-DQB1*	A/T	6.27 × 10^–5^	0.46	1.61 (1.27–2.04)	0.057	0.42	1.27 (0.99–1.62)	2.60 × 10^–5^	1.44 (1.21–1.70)
rs17843614	32,620,661	*HLA-DQA1, HLA-DQB1*,	T/G	8.40 × 10^–5^	0.44	1.59 (1.26–2.01)	0.065	0.42	1.26 (0.99–1.62)	3.59 × 10^–5^	1.43 (1.21–1.69)

Data were obtained using additive logistic regression models with EPACTS based on the Wald test. rs = single-nucleotide polymorphism (SNP) identification number; *Ref* = reference allele, *EA* = effect allele, *MAF* = minor allele frequency, *OR* = odds ratio. In bold, the variant that exceeds the threshold established by Bonferroni correction (*p* = 1.20 × 10^–6^) after the meta-analysis.

**Figure 1:**
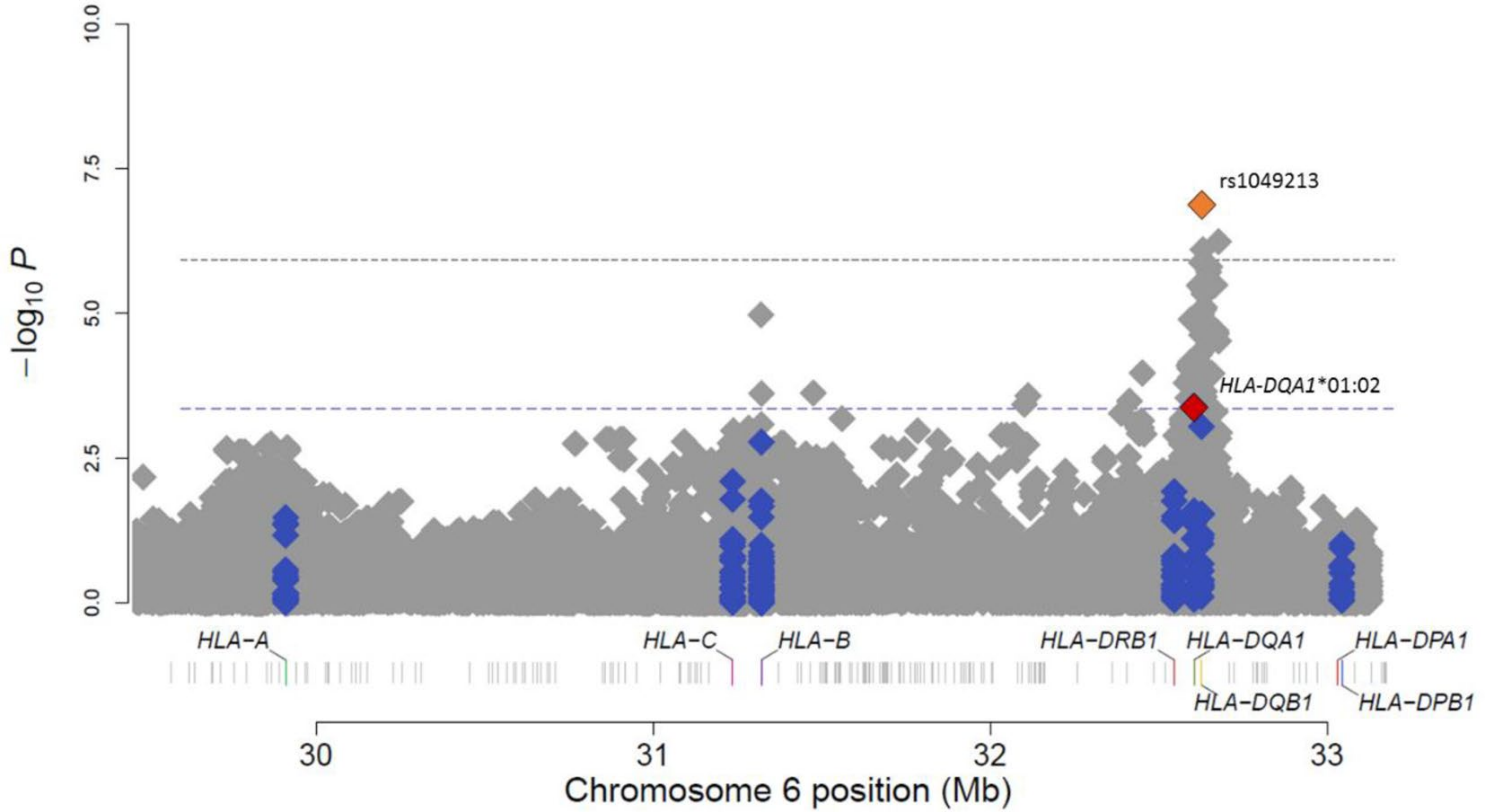
Manhattan plot of meta-analysis results for the association study of chromosome 6 region (grey) and the classical HLA alleles (blue). The y-axis displays transformed *p*-values (–log10) while the x-axis represents chromosome positions (GRCh37/hg19). The horizontal lines correspond to the significance thresholds of each study after Bonferroni correction: the upper for the targeted association (*p* = 1.20 × 10^–6^) and the lower for the classical HLA alleles mapping (*p* = 4.50 × 10^–4^). The significant variants are highlighted in orange (targeted association testing of SNPs) and red (fine mapping of classical HLA alleles). The code used to plot the data was obtained from HATK^30^.

The HLA region is one of the most polymorphic regions of our genome, which makes it difficult to investigate its role in the disease. In this sense, new methods have emerged to impute alleles of classical HLA genes (“classical alleles” from now on) and amino acid polymorphisms from SNP genotyping data^27–29^, providing additional information that can be used to assess the role of this region in asthma physiopathology. We fine mapped this region using a specific method to impute a total of 172 common HLA classical alleles from 3 class I genes (-A, -B, -C) and four class II genes (-DPB1, -DQA1, -DQB1, -DRB1) and assessed their association with asthma susceptibility (Supplementary Table S2). A total of 10 classical alleles within six different HLA genes showed a nominal significance with asthma in stage 1 (*p* < 0.05) (Table 3). Two of them, HLA-DQA1*01:02 and HLA-DQB1*06:04, showed nominal significance and consistent direction of effects in stage 2 (*p* = 0.007 and *p* = 0.025, respectively). The association with asthma protection of HLA-DQA1*01:02 reached study-wise significance in the meta-analysis results from stage 1 and stage 2 (OR [95% CI] = 0.64 [0.50–0.82], *p* = 3.98 × 10^–4^) (Table 3**, **Fig. 1). This association was robust to the model adjustments for sex, age, body mass index, and local NAF or SSA ancestry (Supplementary Table S3) which suggests a correlation between both *HLA-DQA1* and *HLA-DQB1* variants. Accordingly, HLA haplotype analyses revealed that the haplotype DQA1*01:02-DQB1*06:04 was significantly associated with asthma protection (meta-analysis *p* = 4.71 × 10^–4^, OR[95% CI] = 0.47[0.29–0.73]) after setting a Bonferroni threshold at *p* = 2.17 × 10^–3^ (based on the 23 *DQA1*-*DQB1* haplotypes tested, Table 4). Interestingly, HLA-DQB1*06:04 was the allele with the second most significant association in our study.

**Table 3:** Classical HLA alleles nominally significant in stage 1 and their results in stage 2 and in the meta-analysis.

Classical allele	Stage 1			Stage 2			Meta-analysis	
	*p value*	Freq	OR (97.5%)	*p value*	Freq	OR (97.5%)	*p value*	OR (95%)
HLA-DQA1*01:02	**0.019**	**0.151**	**0.67 (0.48–0.94)**	**0.007**	**0.133**	**0.60 (0.41–0.87)**	**3.98 × 10**^**–4**^	**0.64 (0.50–0.82)**
HLA-DQB1*06:04	0.014	0.044	0.45 (0.24–0.85)	0.025	0.032	0.41 (0.19–0.90)	8.98 × 10^–4^	0.44 (0.27–0.71)
HLA-B*07:02	0.003	0.057	0.41 (0.23–0.73)	0.117	0.064	0.65 (0.38–1.12)	1.66 × 10^–3^	0.52 (0.33–0.82)
HLA-C*07:02	0.022	0.058	0.55 (0.33–0.91)	0.143	0.067	0.69 (0.41–1.14)	7.93 × 10^–3^	0.61 (0.43–0.88)
HLA-DRB1*13:02	0.043	0.066	0.55 (0.31–0.98)	0.194	0.048	0.65 (0.34- 1.25)	0.017	0.59 (0.39–0.91)
HLA-DQA1*01:03	0.047	0.102	0.679 (0.46–0.99)	0.267	0.094	0.78 (0.50–1.21)	0.026	0.72 (0.54–0.96)
HLA-DQB1*02:01	0.024	0.108	1.50 (1.06–2.13)	0.446	0.111	1.16 (0.79–1.70)	0.029	1.33 (1.03–1.73)
HLA-DQA1*05:01	0.031	0.108	1.47 (1.04–2.10)	0.502	0.114	1.14 (0.78- 1.67)	0.041	1.31 (1.01–1.70)
HLA-A*31:01	0.037	0.018	0.34 (0.13–0.94)	0.347	0.023	0.68 (0.31–1.52)	0.042	0.52 (0.28–0.98)
HLA-C*01:02	0.024	0.027	0.44 (0.22–0.90)	0.530	0.039	1.23 (0.65–2.33)	0.185	0.74 (0.27–2.03)

Data were obtained using additive logistic regressions models with HIBAG. Freq = classical allele frequency on cases and controls; OR = odds ratio. In bold, the classical allele that exceeds the threshold established by Bonferroni correction (*p* = 4.50 × 10^–4^) after the meta-analysis.

**Table 4:** Results of the HLA haplotype assessment after meta-analysis.

Haplotypes DQA1 ~ DQB1	OR (95%)	*p* value
01:02 ~ 06:04	**0.47 (0.29–0.73)**	**4.71 × 10**^**–4**^
05:01 ~ 02:01	1.36 (1.07–1.73)	9.66 × 10^–3^
01:02 ~ 06:09	0.47 (0.23–0.92)	0.021
01:02 ~ 06:02	0.74 (0.52–1.05)	0.081
03:03 ~ 03:01	1.44 (0.88–2.36)	0.123
03:03 ~ 04:02	0.54 (0.19–1.33)	0.157
01:03 ~ 06:01	0.47 (0.11–1.49)	0.168
04:01 ~ 04:02	0.72 (0.43–1.19)	0.184
01:05 ~ 05:01	1.35 (0.74–2.45)	0.287
05:05 ~ 03:01	1.11 (0.91–1.37)	0.289
01:03 ~ 06:03	0.88 (0.67–1.15)	0.323
01:02 ~ 05:02	1.23 (0.76–1.95)	0.368
01:02 ~ 05:01	1.48 (0.53–4.06)	0.395
02:01 ~ 02:02	1.10 (0.87–1.38)	0.424
03:03 ~ 03:02	1.20 (0.73–1.94)	0.447
02:01 ~ 03:03	0.83 (0.48–1.42)	0.480
06:01 ~ 03:01	1.64 (0.30–8.82)	0.481
03:02 ~ 03:03	0.70 (0.12–3.08)	0.605
01:02 ~ 05:04	0.65 (0.06–4.00)	0.609
01:01 ~ 05:01	1.06 (0.84–1.33)	0.612
03:01 ~ 03:02	0.95 (0.72–1.26)	0.726
01:04 ~ 05:03	1.09 (0.59–2.00)	0.761
01:02 ~ 06:03	1.23 (0.18–7.27)	0.788

Association test based on a chi-square test. *CI* = confidence interval, *OR* = odds ratio. In bold, the haplotype that exceeds the threshold established after Bonferroni correction (*p* = 2.17 × 10^–3^).

The HLA-DQA1*01:02 sequence translation to the amino acid sequence revealed a specific missense substitution within exon 2 at position 57 (Glu57Gln, E57Q) (Supplementary Figure S2). Mapping each nucleotide position within the altered codon indicated that E57Q corresponds to rs10093, which did not reach statistical significance in our study (meta-analysis OR [95%CI] = 1.13 [0.96–1.33], *p* = 0.139). However, a few of its linkage disequilibrium (LD) proxies (r^2^ > 0.77 in Europeans) were nominally significant in the meta-analysis (results for the leading variant rs9271588, OR [95% CI] = 1.20 [1.02–1.41], *p* = 0.027). Bioinformatic tools for variant prioritization (DSNetwork, VEP, and RegulomeDB) showed that the top-ranked proxy for rs10093 was rs9271588, an intergenic variant located between *HLA-DQA1* and *HLA-DRB1*, which was predicted to have the higher confidence of regulatory impact (Supplementary Table S4). In this sense, rs9271588 was found to be linked to relevant functional consequences based on diverse in silico approaches (Supplementary Table S4). As a summary, this SNP is located within regulatory elements of genes in a subset of tissues and cell types, including enhancer and promoter histone marks and DNase I hypersensitive sites (Supplementary Table S4). Furthermore, rs9271588 is also implicated in disrupting regulatory motifs and protein binding (Supplementary Table S4). Hi-C experiment results supported physical chromatin interactions between the region harbouring rs9271588 and *HLA-DQB1* and *HLA-DRB1* promoter regions in diverse cell lines (Supplementary Table S4). Additionally, GTEx data supported that rs9271588 is an eQTL and sQTL for *HLA-DQA1*, *HLA-DQB1*, and *HLA-DRB1* in different tissues, including lung, esophagus, and whole blood (Supplementary Table S4). This SNP also has high CellulAr dePendent dEactivating (CAPE) scores for eQTLs for *HLA-DQA1*, *HLA-DQB1*, and *HLA-DRB1* in a lymphoblastoid cell line (Supplementary Table S4). Finally, transcriptomic data from bronchial brushing and bronchoalveolar lavage (BAL) samples revealed that *HLA-DQA1* (only available for bronchial brushing), *HLA-DQB1*, and *HLA-DRB1* were downregulated in patients with severe asthma compared to healthy controls (Supplementary Figure S3 and Supplementary Figure S4).

## Discussion

Several studies support that non-European populations would carry the greatest burden of asthma^10^. However, the assessed genetic diversity of the published GWAS of asthma is strongly biased towards Central and Northern Europeans, and additional genetic studies in more diverse populations are required to identify novel genetic risk factors for asthma^10^. Our study provides significant insights of genetics underlying asthma susceptibility in a population with the largest recent African admixture recorded so far among southwestern Europeans^31^. Besides, the available estimates support that the Canary Islands population has the greatest prevalence of asthma in Spain^23,24^. Here we performed a targeted staged association study of five genomic regions that we previously described to be enriched in African ancestry among Canary Islanders, and revealed that genetic variants in 6p22.3–p21.32 and within *HLA-DQA1* and *HLA-DQB1* were significantly associated with asthma risk. HLA genes are located within the MHC and encode a group of proteins with important functions in cell–cell interactions and are critical regulators of the immune response^32^. In fact, a number of genetic variants from the MHC (some of them linked to *HLA-DQA1* and *HLA-DQB1*) have been associated with asthma in large genetic association studies^10,33,34^. Interestingly, other studies in populations with African ancestry have allowed to link classical HLA class II gene alleles to the total serum IgE levels in patients with asthma^35,36^.

Our staged association study revealed the association of rs1049213 with asthma risk, which has been previously linked to asthma, supporting the robustness of our results. Accordingly, it is broadly known that interpreting SNP associations can be problematic, partly due to the difficulty in identifying the causal variant^37^, and this interpretation becomes even more complicated when the prioritized variant is in the highly polymorphic HLA region. Because of that, classical HLA alleles are more biologically informative and can be related to stronger effects than individual SNPs^38,39^. Our results revealed that the classical HLA allele HLA-DQA1*01:02 was significantly associated with asthma protection. HLA-DQA1*01:02 had never been linked to asthma risk before, although it has been related with protection from infectious^40^ and autoimmune diseases, including type-1 diabetes^41^, Crohn’s disease^42^, tubulointerstitial nephritis, and uveitis syndrome^43^, and risk for peanut allergy^44^. Additionally, HLA haplotype analyses revealed, for the first time, the association of DQA1*01:02-DQB1*06:04 with asthma protection, which supports that the combination of variants within both genes could influence the pathophysiology of the disease. This is in line with previous studies in asthma where HLA haplotypes were identified to be significantly associated with the disease^45^. The protein encoded by *HLA-DQA1* binds to the protein encoded by *HLA-DQB1*, constituting a heterodimer that plays a central role in the immune system. Interestingly, our analyses prioritized an LD proxy of the missense change defining the HLA-DQA1*01:02 as a clear lung eQTL for *HLA-DQA1* and *HLA-DQB1*. This evidence agrees with human transcriptomic observations revealing a reduction of the *HLA-DQA1* and *HLA-DQB1* lung gene expression in patients with severe asthma compared to healthy controls. Thus, a lower activity of the heterodimer among asthma patients would be expected in carriers of this classical HLA allele.

We acknowledge some limitations of this study. First, all individuals were selected based on their self-declaration of having two generations of ancestors born in the Canary Islands. Nevertheless, this agrees with the National Institutes of Health (NIH) guidelines, and previous genetic studies in the same population have shown that this selection has no major effect on the findings of the study^16,22^. Additionally, the use of population controls could bias the results in the sense that it is possible that these individuals could develop asthma or related phenotypes during their lives. However, this type of controls has been widely used in genetic and genomic studies and is the most optimal control group to get the most representative subset of the population, reducing the selection bias^10,46^. We did not include other confounders such as environmental factors or responses to asthma treatment because data were lacking for most individuals. However, we validated our results in an independent case–control sample, supporting that our results may not be biased by these variables. Significant differences in sex and age were found among cases and controls from both stages, although results remained significant after including these variables in the models. Finally, in addition to the difficulties inherent to the highly polymorphic HLA region, because of technical limitations (e.g., array content and imputation algorithm), we did not assess all common classical HLA alleles of this population, which could have masked additional asthma associations.

## Conclusions

A two-stage association study targeting the genomic regions with an excess of African ancestry in the Canary Islands population revealed a novel classical HLA allele (HLA-DQA1*01:02) associated with asthma protection. This suggests that not all shared genetic risks between asthma and autoimmune diseases have opposite directions of effect^47^. Further studies will be needed to validate these results in other populations, as well as to assess the potential of HLA-DQA1*01:02 as an asthma biomarker.

## Methods

### Study design and participants

The study was performed in accordance with The Code of Ethics of the World Medical Association (Declaration of Helsinki) and approved by the Research Ethics Committee from the Hospital Universitario Nuestra Señora de Candelaria. Written informed consents were collected from all subjects or their representatives.

We performed a two-stage case–control study of asthma susceptibility targeting the five genomic regions that showed an excess of African ancestry in the current population of the Canary Islands (chr2:133,952,040–144,266,489; chr3:10,539,482–11,710,471; chr3:177,443,968–178,679,751; chr6:24,703,442–36,288,651; and chr13:57,962,413–70,091,195)^22^. The stage 1 was performed to prioritize genetic variants within these regions, while the stage 2 allowed us to validate these associations in an independent data set. A meta-analysis combining stage 1 and stage 2 association results was performed to establish the genetic variants associated with asthma in this study.

All case and control individuals declared at least two generations of ancestors born in the Canary Islands (Spain). The stage 1 consisted of 314 cases of asthma and 674 controls, while the stage 2 comprised a total of 278 asthma patients and 349 controls. All asthma patients were part of the Genetics of Asthma study in the Spanish population (GOA) study^48^ and were diagnosed according to the Global Initiative for Asthma (GINA) guidelines^49^. Population controls were obtained from the Cardiovascular, Diabetes, and Cancer (CDC) cohort study from the Canary Islands^50^. DNA was extracted from peripheral blood following column-based methods. Further details of these studies can be found elsewhere^16,22,51^.

### Genotyping and statistical analyses

**Figure 2:**
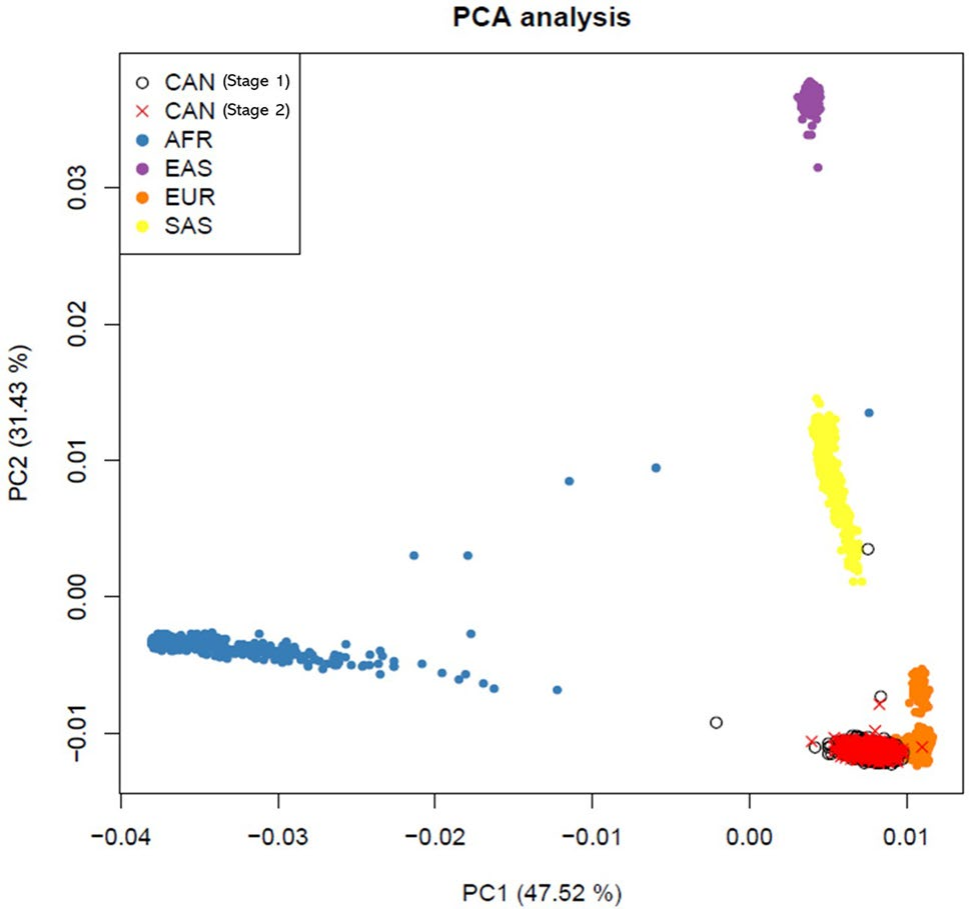
Plot of the first two principal components (explaining 78.95% of variability) of the individuals analysed in stage 1 and 2, projected on data of African (AFR), East Asian (EAS), European (EUR), and South Asian (SAS) populations from The 1000 Genomes Project.

All DNA samples were subject to genotyping of 587,352 SNPs using the Axiom Genome-Wide Human CEU 1 Array (Thermo Fisher Scientific, Waltham, MA, USA) by the Spanish Genotyping Center (CeGen). Variant calling was performed separately in cases and controls from the discovery study and low-quality SNPs and samples were excluded according to the manufacturer’s instructions. Stringent quality controls were subsequently conducted using R programming (v3.2.2)^52^ and PLINK v1.9^53^. Genetic variants with genotype call rates (CR) < 95%, low minor allele frequency (MAF < 5%), or that deviated from Hardy Weinberg equilibrium (HWE, *p* < 1.0 × 10^−6^) were excluded. A total of 403,615 filtered high-quality SNPs were kept for downstream analyses. Additionally, we also excluded individuals with missing clinical information, sex mismatches between records and those inferred from the genotype data, CR < 95%, high degree of kinship with others included in the study (PIHAT > 0.2), or that constituted heterozygosity outliers. A principal component analysis (PCA) was performed using PLINK v1.9^53^ to obtain the leading principal components (PCs) to correct for population stratification during association testing. The PCA was assessed for stages 1 and 2, separately, and individuals were projected on data from The 1000 Genomes Project^54^ (**Fig. ****2**).

Variant imputation of chromosomes 2, 3, 6, and 13 was then conducted on filtered data using the Michigan Imputation Server^55^ selecting SHAPE-IT v2.r790 for chromosome phasing^56^ and European population data from the Haplotype Reference Consortium release 1.1 as reference panel^57^. Logistic regressions were performed with EPACTS v3.2.6^58^ using a binary Wald test and assuming an additive inheritance model. We included the first four PCs as covariates. Variants with MAF < 1% and a poor imputation quality (Rsq < 0.3) were excluded from the analysis. The genomic inflation factor (λ) of the results was calculated with the R package “qqman”^59^ (Supplementary Figure S5).

Genotyping and statistical analyses of the data from the two stages followed the same procedures. To assess the overall effect size of associated SNPs across the two stages, a meta-analysis was conducted using METASOFT v2.0.1, where effect heterogeneity was assessed with the Cochran's Q test significance^60^. Only variants showing the same direction of effects on both studies were considered. We estimated the effective number of independent tests with the Genetic type 1 error calculator (GEC)^61^ and established the meta-analysed significance at *p* = 1.20 × 10^–6^ based on Bonferroni correction.

### Fine mapping of the HLA region

A more detailed assessment of the HLA region, residing in chromosome 6, was performed on both stages separately. Classical HLA alleles from three class I genes (-A, -B, -C) and four class II genes (-DPB1, -DQA1, -DQB1, -DRB1) were imputed with HLA Genotype Imputation with Attribute Bagging (HIBAG) v1.4 using imputation models adapted to the Axiom Genome-Wide Human CEU 1 Array and a European reference panel^27^. The association analysis with asthma was also performed with HIBAG using an additive model on those individuals with a high confidence score (probability threshold > 0.5). The four first PCs were included in the models. A meta-analysis was conducted with METASOFT v2.0.1^60^ for the common alleles (MAF ≥ 1%) that showed a nominal association (*p* < 0.05) and the same direction of effects on both stages. For this study, a meta-analysed significance threshold was declared at *p* = 4.50 × 10^–4^ after Bonferroni correction based on the number of alleles tested. A post-hoc sensitivity analysis was also performed to address potential biases of the results including sex, age, and local ancestry estimates as covariables in the HIBAG association models. The estimation of local ancestry was carried out using ELAI^62^ assuming a three-way admixture as detailed elsewhere (European, NAF, and SSA)^16,22^. HLA haplotype analyses were performed using BIGDAWG^63^, focusing on those genes that were in the vicinity of the variants that showed significant association*.* Statistical testing of the haplotype difference was based on a Chi-squared test, using BIGDAWG’s default parameters.

### Functional annotation of variants and gene expression

We used HIBAG to convert the associated P-coded HLA alleles to amino acid sequences, in order to reveal amino acid residues and, hence, those SNPs that may explain the classical HLA allele associations. We explored the potential biological consequences of the SNP predicting the altered codon in the amino acid in the classical HLA allele and its best proxies (i.e., in strong LD in Europeans, r^2^ > 0.7) by using different in silico tools. Variant prioritisation was based on results obtained with DSNetwork^64^, RegulomeDB^65^, and VEP^66^. Additionally, we assessed the potential regulatory role of the variants using HaploReg v4.1^67^ and RegulomeDB, as well as the existence of long-distance physical interactions with Capture Hi-C Plotter^68^. We also accessed GTEx^69^, ExSNP^70^, and SNPdelScore^71^ to evaluate tissue-specific local expression quantitative trait loci (eQTLs) and splicing quantitative trait loci (sQTLs). Further details are provided in the Supplement.

In parallel, we accessed the results of public gene expression studies of asthma available in Gene Expression Omnibus (GEO). Differential gene expression between cases with asthma and healthy controls was examined for those genes in the vicinity of the SNPs and classical HLA alleles significantly associated with asthma susceptibility in our study, to provide additional information on the role of these genes in asthma physiopathology. First, we accessed transcriptomic data of bronchial brushing samples from 27 healthy controls and 128 individuals with asthma (72 non-severe asthma, 56 severe asthma) (GSE63142)^72^. Then, we accessed gene expression results of BAL samples from 12 healthy controls and 74 asthmatic individuals (28 non-severe asthma, 46 severe asthma) (GSE74986)^73^. The differential expression was assessed with two-sample t-tests and one-way analyses of variance (ANOVA). Further details are provided in the Supplement.

## Supplementary Information

Supplementary Information.

## Data Availability

The data that support the findings of this study are available on request from the corresponding author. The genotype and sequence data are not publicly available due to privacy or ethical restrictions.
