# Supplementary material for: Targeted analysis of genomic regions enriched in African ancestry reveals novel classical HLA alleles associated with asthma in Southwestern Europeans
Source: Sci Rep. 2021 Dec 8;11:23686. doi: 10.1038/s41598-021-02893-w (PMC8654850; doi:10.1038/s41598-021-02893-w)
Supplement: Supplementary file 1 — Supplementary Information. [file 41598_2021_2893_MOESM1_ESM.pdf]

SUPPLEMENTARY MATERIAL

Targeted analysis of genomic regions enriched in African ancestry reveals novel classical HLA alleles associated with asthma in Southwestern Europeans

Eva Suarez-Pajes, Claudio Díaz-García, Héctor Rodríguez-Pérez, Jose M. Lorenzo-Salazar, Itahisa Marcelino-Rodríguez, Almudena Corrales, Xiuwen Zheng, Ariel Callero, Eva Perez-Rodriguez, Jose C. Garcia-Robaina, Rafaela González-Montelongo, Carlos Flores, Beatriz Guillen-Guio

Functional annotation of variants and gene expression

We explored the potential biological consequences of the single nucleotide polymorphism (SNP) predicting the altered codon in the amino acid in the classical HLA allele and its best proxies (i.e., in strong linkage disequilibrium [LD] in Europeans, $r^2>0.7$) by using different in silico tools, with the aim of providing additional information on the role of HLA class II gene dysregulation in asthma. Variant prioritisation was based on functional scores obtained with DSNetwork [1] and RegulomeDB [2], allowing to identify the most probable functional variants. We used Ensembl Variant Effect Predictor (VEP) to determine the consequences of top variants in genes, protein sequence, and regulatory regions [3]. Additionally, we assessed the potential regulatory role of the variants using HaploReg v4.1 [4] and RegulomeDB, including the evaluation of chromatin states by identifying histone marks, DNase I hypersensitive sites, and altered regulatory motifs in that region. We used Capture Hi-C Plotter [5] to analyse the existence of long-distance physical chromatin interactions with regulatory elements and gene promoters in different tissues, considering a default score of 5 as threshold to identify PCHi-C interactions . We also accessed GTEx [6], ExSNP [7], and SNPdelScore [8] to evaluate tissue-specific local expression quantitative trait loci (eQTLs) and splicing quantitative trait loci (sQTLs). We reported QTL associations at $p\leq0.05$.

In parallel, we accessed the results of two public gene expression studies of asthma that were catalogued in Gene Expression Omnibus (GEO). First, we accessed transcriptomic data of samples from 27 healthy controls and 128 individuals with asthma (Ref. GSE63142) [9]. These samples were obtained from the Severe Asthma Research Program (SARP) [10] and the RNA was extracted from bronchial epithelial cells. Patients were classified according to their phenotype in 72 non-severe and 56 severe asthma patients. Then, we accessed gene expression results of bronchoalveolar lavage (BAL) samples from 12 healthy controls and 74 asthmatic individuals (28 non-severe asthma, 46 severe asthma) (GSE74986) [11]. Samples from healthy controls and patients with moderate asthma are from the Study of the Mechanisms of Asthma (MAST, ClinicalTrials.gov:NCT00595153), while those samples from patients with severe asthma are from the BOBCAT study [12]. For both transcriptomic studies (GSE63142 and GSE74986), expression arrays were used to obtain the gene expression profiles. The differential gene expression between cases with asthma and healthy controls was examined using shinyGEO [13] and R programming [14] for those genes in the vicinity of the SNPs and classical HLA alleles significantly associated with asthma susceptibility in our study (i.e. *HLA-DQA1* and *HLA-DQB1*). We also included *HLA-DRB1* in analyses since the best ranked proxy (rs9271588) for rs10093 is an intergenic variant located between *HLA-DQA1* and *HLA-DRB1*. We assessed the average

intensity differences of the matrix probes targeting *HLA-DQA1*, *HLA-DQB1*, and *HLA-DRB1* using two-sample t-tests and one-way analyses of variance (ANOVA) (**Figures S3-4**).

Figures

Figure S1. Regional plot of the meta-analysed association results of the chromosome 6 region indicating the significant single-nucleotide polymorphism of the study (rs1049213). The y-axis displays transformed *p*-values (–log10) while the x-axis represents chromosome positions (GRCh37/hg19). The results for the remaining SNPs are represented with a colour code representing the degree of linkage disequilibrium with rs1049213 based on pairwise r2 values in European populations of The 1000 Genomes Project**.** Results were plotted using LocusZoom (http://locuszoom.org/).

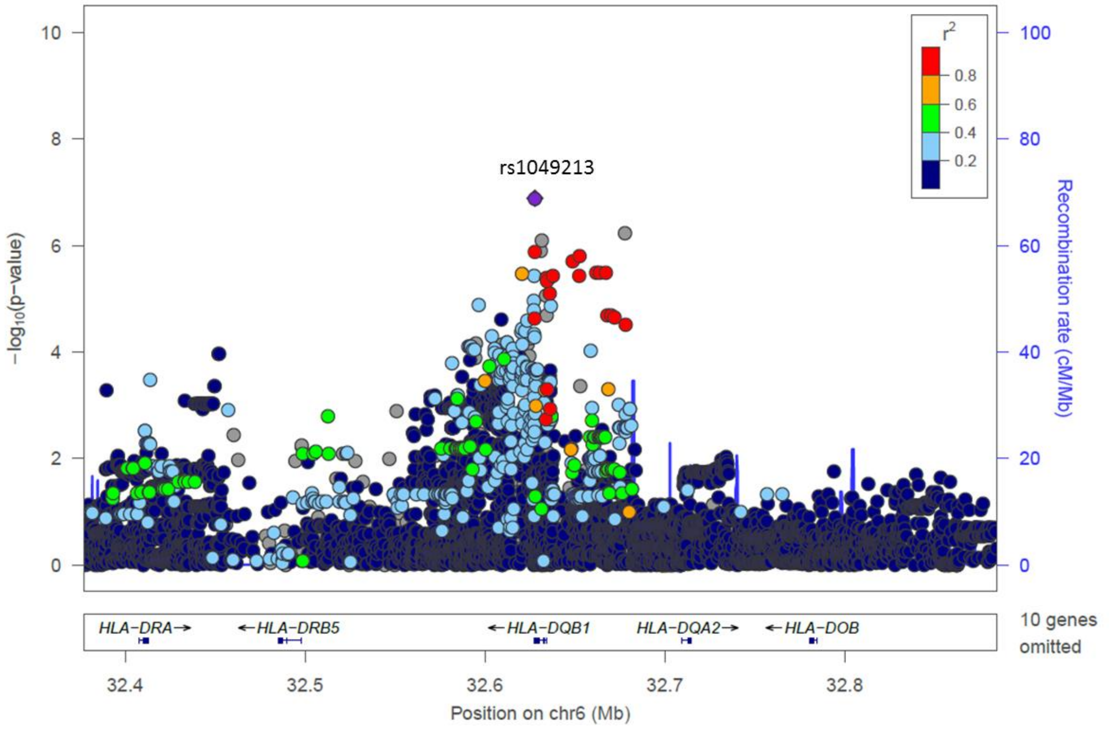

Figure S2. Partial predicted amino acid sequence of exon 2 from *HLA-DQA101:02 indicating the E57Q change.** An alignment between the reference protein sequence (top) starting at codon 28 and those from the other classical alleles included in the p-code.

```
X01.02
              ADHVASCGVNLYQFYGPSGQYTHEFDGDEEFYVDLERKETAWRWPEFSKFGGFDPQGALRNMAVAKHNLNIMIKRYNSTAATNE
01:02:01:01   -----------------------------Q------------------------------------------------------
01:02:01:02   -----------------------------Q------------------------------------------------------
01:02:01:03   -----------------------------Q------------------------------------------------------
01:02:01:04   -----------------------------Q------------------------------------------------------
01:02:02      -----------------------------Q------------------------------------------------------
01:02:03      -----------------------------Q------------------------------------------------------
01:02:04      -----------------------------Q------------------------------------------------------
01:08         *----------------------------Q------------------------------------------------------
01:09         *----------------------------Q------------------------------------------------------
01:11         -----------------------------Q------------------------------------------------------
```

Figure S3. Gene expression of (a) *HLA-DQA1*, (b) *HLA-DQB1*, and (c) *HLA-DRB1* in 27 healthy controls, 72 individuals with non-severe asthma, and 56 with severe asthma obtained from transcriptomic data from bronchial brushing samples. Expression differences were assessed using ANOVA followed by t-tests. The probes used for each of the genes are indicated in parentheses in the title. Data obtained from the GEO accession GSE63142.

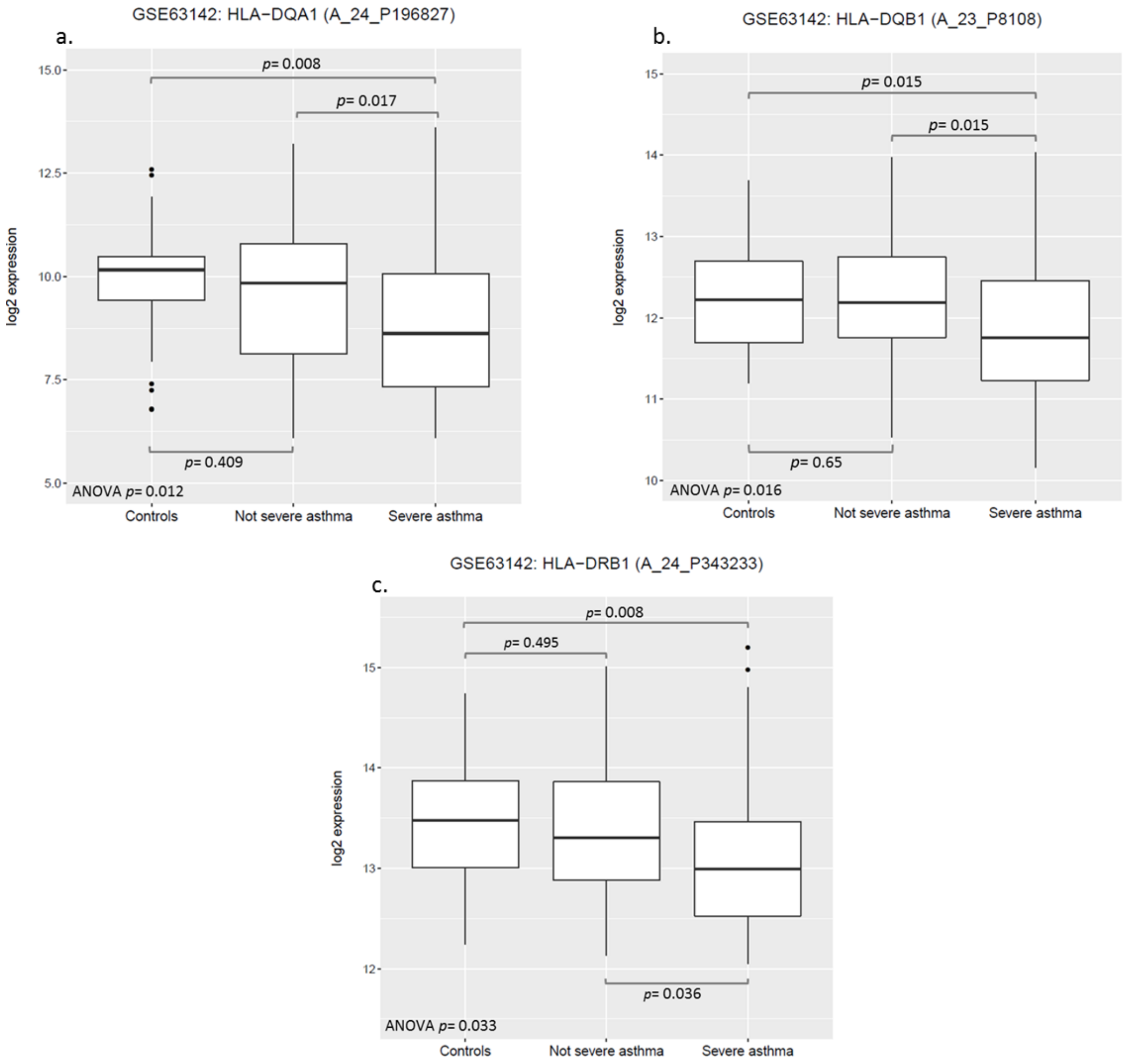

Figure S4. Gene expression of (a) *HLA-DQB1* and (b) *HLA-DRB1* in 12 healthy controls, 28 individuals with non-severe asthma, and 46 with severe asthma obtained from transcriptomic data from bronchoalveolar lavage (BAL) samples. Expression differences were assessed using ANOVA followed by t-tests. The probes used for each of the genes are indicated in parentheses in the title. Data obtained from the GEO accession GSE74986.

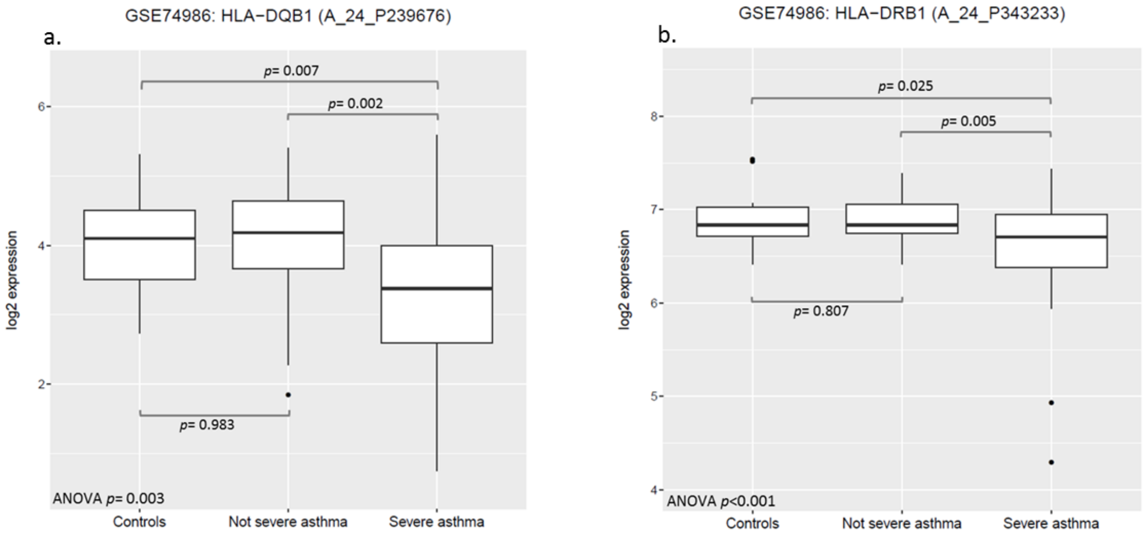

Figure S5. Quantile-Quantile (Q-Q) plot. Representation of observed (y-axis) *vs* expected (x-axis) -log10 *p*-values for the first stage of the association study. The genomic inflation factor (λ=1.074) did not indicate inflation of the results.

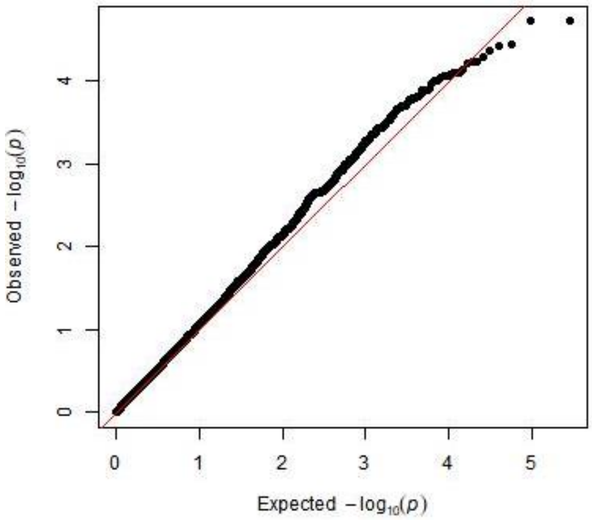

Tables

Table S1. Total number of filtered variants tested in the targeted association analysis for each of the chromosomal regions with an excess of African local ancestry.

Chromosome	Chromosomal region	Total variants analysed
2	2q21.2-q22.3	31,760
3	3p25.3 and 3q26.32	7,179
6	6p22.3-p21.32	63,475
13	13q21.1-q21.33	38,541

Table S2. Total number of classical alleles tested with HIBAG for each of the classical HLA genes.

Genes	Common classical alleles
HLA-A	26
HLA-B	45
HLA-C	23
HLA-DRB1	31
HLA-DQA1	14
HLA-DQB1	16
HLA-DPB1	17

Table S3. Sensitivity analysis for the *HLA-DQA1*01:02* allele association with asthma in the two stages after meta-analysis.

	OR [95% CI]	*p-value*
**Unadjusted***	0.64 [0.50-0.82]	3.98×10^{-4}
**Sex**	0.65 [0.50-0.83]	6.16×10^{-4}
**Age**	0.58 [0.43-0.78]	3.57×10^{-4}
**Sex and age**	0.59 [0.43-0.80]	5.72×10^{-4}
**BMI****	0.65 [0.48-0.87]	3.82×10^{-3}
**Local ancestry**		
**NAF**	0.62 [0.48-0.80]	2.36×10^{-4}
**SSA**	0.62 [0.48-0.79]	1.77×10^{-4}

*Data adjusted only for the first four principal components. **Missing data for 20% of individuals. BMI = Body Mass Index, NAF= North-African, SSA= Sub-Saharan African.

Table S4. Functional analysis of rs10093 and its best linkage disequilibrium proxy.

	rs10093	rs9271588
**Chromosome location**	chr6:32609173	chr6:32590953
**r2**	-	0.77
**Genomic context**	*HLA-DQA1* (exon 2)	Intergenic to *HLA-DQA1* and *HLA-DRB1*
**VEP**	Missense variant	Regulatory region variant
**regulomeDB (rank)**	TF binding + DNase peak (4)	eQTL + TF binding + any motif + DNase Footprint + DNase peak (1b)
**regulomeDB (score)**	0.61	0.99
**Enhancer histone marks [HaploReg]**		
H3K4me1	Blood & T-cell, HSC & B-cell, Heart, Digestive, Lung, Liver, BLD.GM12878, BLD.CD14.MONO	ESC, iPSC, Blood & T-cell, HSC & B-cell, Epithelial, Thymus, Adipose, Sm. Muscle, Digestive, Spleen, BLD.DND41.CNCR, BLD.GM12878, BRST.HMEC, BLD.CD14.MONO, SKIN.NHEK
H3K27ac	Blood & T-cell, HSC & B-cell, Digestive, Lung, BLD.GM12878	Blood & T-cell, HSC & B-cell, Thymus, Muscle, Digestive, Liver, Spleen, BLD.DND41.CNCR, BLD.GM12878, BRST.HMEC, BLD.CD14.MONO
**Promoter histone marks [HaploReg]**		
H3K4me3	ES-deriv, Blood & T-cell, HSC & B-cell, Brain, Adipose, Digestive, BLD.GM12878, BLD.CD14.MONO	Blood & T-cell, HSC & B-cell, Digestive, BLD.GM12878, BLD.CD14.MONO, BRN.NHA, SKIN.NHEK
H3K9ac	Liver, BLD.GM12878	Digestive, Liver, BLD.GM12878, BLD.CD14.MONO
**DNAse [HaploReg]**	BLD.GM12878	ES-deriv, Blood & T-cell, HSC & B-cell, Epithelial, Thymus, Muscle, Digestive, BLD.GM12878, BRST.HMEC, MUS.HSMM, BLD.CD14.MONO, SKIN.NHEK
**Altered regulatory motifs [HaploReg]**	HDAC2	Cdx2, Pdx1
**Proteins bound [HaploReg]**	POL2, POL24H8	BCL11A, MEF2A, MEF2C, NFKB, OCT2, POU2F2, RFX5, SP1, TBP
**CHICP***		
Blood cells	*HLA-DRA*	*AGPAT1*, *ATF6B*, *BRD2*, *BTNL2*, *DPCR1*, *HLA-DMA*, *HLA-DMB*, *HLA-DOB*, *HLA-DPA1*, *HLA-DPB*, *HLA-DPB1*, *HLA-DQA2*, *HLA-DQB1*, *HLA-DRA*, *HLA-DRB1*, *KIFC1*, *NELFE*, *PSMB8*, *PSMB9*, *RNF5*, *TAP1*, *TAP2*, *TNXB*
CD34/GM12878	*HLA-DQB1*, *HLA-DRA*, *HLA-DRB1*	None
Pancreatic Islets	None	*BTNL2*, *HLA-DOB*, *HLA-DQB1*, *HLA-DRA, PSMB9*, *TAP1*, *TAP2*
hESC Derived Cardiomyocytes	None	*DUSP22, HLA-DOB, HLA-DQB1, HLA-DRA, HLA-DRB1, PSMB9, TAP1, TAP2*
**eQTLs [GTEX]** Tissue-specific $P \leq 0.05$	*Lymphoblastoid cell lines (HLA-DRB1, HLA-BRB5)*	Cells - Cultured fibroblasts (*HCG23*, *LY6G5B*, *PBX2*); Cells - EBV-transformed lymphocytes (*HLA-DQA1*, *HLA-DQA2*, *HLA-DRB1*, *HLA-DRB6*); Esophagus (*C4A*, *CYP21A1P*, *HCG23*, *HLA-DQA1*, *HLA-DQA2*, *HLA-DQB1*, *HLA-DRA*, *HLA-DRB1*, *HLA-DRB6*, *LY6G5B*, *PRRT1*, *STK19B*); Lung (*HCG23*, *HLA-DQA1*, *HLA-DQA2*, *HLA-DQB1*, *HLA-DRB1*, *HLA-DRB6*, *MSH5*,

		PRRT1, *STK19B*); Minor Salivary Gland (*HLA-DQA2*, *HLA-DRB6*); Whole Blood (*HLA-DQA1*, *HLA-DQA2*, *HLA-DQB1*, *HLA-DQB2*, *HLA-DRB1*, *HLA-DRB6*, *HLA-DRB9*, *LY6G5B*)
**sQTLs [GTEX]** Tissue-specific *P* ≤ 0.05	None	Esophagus (*HLA-DQA1*, *HLA-DQA2*, *HLA-DQB1*, *HLA-DQB2*, *HLA-DRA*, *HLA-DRB1*, *HLA-DRB5*, *HLA-DRB6*, *STK19B*); Cells - EBV-transformed lymphocytes (*HLA-DQA2, HLA-DRB1*, *HLA-DRB5*, *HLA-DRB6*); Lung (*HLA-DQA2*, *HLA-DQB1*, *HLA-DQB2*, *HLA-DRB1*, *HLA-DRB5*, *HLA-DRB6*); Minor Salivary Gland (*HLA-DQA1*, *HLA-DQA2*, *HLA-DRB1*, *HLA-DRB5*, *HLA-DRB6*); Whole Blood (*HLA-DQA1*, *HLA-DQA2*, *HLA-DQB1*, *HLA-DQB2*, *HLA-DRB1*, *HLA-DRB5*, *HLA-DRB6*) **
**Score CAPE eQTL >0.5 [SNPDelScore]**	None	Lymphoblastoid cell line (*HLA-DQA1*, *HLA-DQA2*, *HLA-DQB1*, *HLA-DRA*, *HLA-DRB1*, *HLA-DRB5*)

BLD.CD14.MONO, Monocytes-CD14+ RO01746 Primary Cells; BLD.DND41.CNCR, Dnd41 TCell Leukemia Cell Line; BLD.GM12878, GM12878 Lymphoblastoid Cells; BRN.NHA, NH-A Astrocytes Primary Cells; BRST.HMEC, HMEC Mammary Epithelial Primary Cells; CAPE, cellular dependent deactivating mutations; CD34, human hematopoietic progenitor cell line; CHiCP, capture HiC plotter; eQTL, expression quantitative; ES, Embryonic stem; ESC, embryonic stem cells; HSC, hematopoietic stem cells; iPSC induced pluripotent stem cells; MUS.HSMM, HSMM Skeletal Muscle Myoblasts Cells; SKIN.NHEK, NHEK-Epidermal Keratinocyte Primary Cells; TF, transcription factor; VEP, Variant Effect Predictor. *Blood cells (Javierre et al dataset), CD34/GM12878 (Misfud et al dataset), Pancreatic Islets (Miguel-Escalada et al dataset), hESC Derived Cardiomyocytes (Choy et al dataset) **only tissues with biological relevance are shown.

References

1. Lemaçon, A. et al. DSNetwork: An Integrative Approach to Visualize Predictions of Variants' Deleteriousness. Front. Genet. 10, 1–9 (2020).

2. Boyle, A. P. et al. Annotation of functional variation in personal genomes using RegulomeDB. Genome Res. 22, 1790–1797 (2012).

3. McLaren, W. et al. The Ensembl Variant Effect Predictor. Genome Biol. 17, 1–14 (2016).

4. Ward, L. D. & Kellis, M. HaploReg: A resource for exploring chromatin states, conservation, and regulatory motif alterations within sets of genetically linked variants. Nucleic Acids Res. 40, 930–934 (2012).

5. Schofield, E. C. et al. CHiCP: A web-based tool for the integrative and interactive visualization of promoter capture Hi-C datasets. Bioinformatics 32, 2511–2513 (2016).

6. Lonsdale, J. et al. The Genotype-Tissue Expression (GTEx) project. Nat. Genet. 45, 580–585 (2013).

7. Yu, C. H., Pal, L. R. & Moult, J. Consensus Genome-Wide Expression Quantitative Trait Loci and Their Relationship with Human Complex Trait Disease. Omi. A J. Integr. Biol. 20, 400–414 (2016).

8. Alvarez, R. V., Li, S., Landsman, D. & Ovcharenko, I. SNPDelScore: combining multiple methods to score deleterious effects of noncoding mutations in the human genome. Bioinformatics 34, 289–291 (2018).

9. Modena, B. D. et al. Gene expression in relation to exhaled nitric oxide identifies novel asthma phenotypes with unique biomolecular pathways. Am. J. Respir. Crit. Care Med. 190, 1363–1372 (2014).

10. Moore, W. C. et al. Identification of Asthma Phenotypes Using Cluster Analysis in the Severe Asthma Research Program. Am. J. Respir. Crit. Care Med. 181, 315–323 (2010).

11. Sun, Y. et al. Inhibition of the kinase ITK in a mouse model of asthma reduces cell death and fails to inhibit the inflammatory response. Sci. Signal. 8, 1–14 (2015).

12. Jia, G. et al. Periostin is a systemic biomarker of eosinophilic airway inflammation in asthmatic patients. J. Allergy Clin. Immunol. 130, 647-654.e10 (2012).

13. Dumas, J., Gargano, M. A. & Dancik, G. M. ShinyGEO: A web-based application for analyzing gene expression omnibus datasets. Bioinformatics 32, 3679–3681 (2016).

14. Bunn, A. & Korpela, M. R: A language and environment for statistical computing. 2, 1–12 (2016).
